# Neuregulin 1 Type I Overexpression Is Associated with Reduced NMDA Receptor–Mediated Synaptic Signaling in Hippocampal Interneurons Expressing PV or CCK

**DOI:** 10.1523/ENEURO.0418-17.2018

**Published:** 2018-05-08

**Authors:** Dimitrios Kotzadimitriou, Wiebke Nissen, Melinda Paizs, Kathryn Newton, Paul J. Harrison, Ole Paulsen, Karri Lamsa

**Affiliations:** 1 Department of Pharmacology, University of Oxford, Oxford, OX1 3QT, UK; 2 Department of Physiology, Anatomy and Neuroscience, University of Szeged, Szeged, 6720, Hungary; 3 Department of Psychiatry, University of Oxford, and Oxford Health NHS Foundation Trust, Oxford, UK; 4 Department of Physiology, Development and Neuroscience, University of Cambridge, Cambridge, UK

**Keywords:** Axo-axonic cell, basket cell, cholecystokinin, ErbB4 receptor, NMDA receptors, parvalbumin, schizophrenia

## Abstract

Hypofunction of *N*-methyl-d-aspartate receptors (NMDARs) in inhibitory GABAergic interneurons is implicated in the pathophysiology of schizophrenia (SZ), a heritable disorder with many susceptibility genes. However, it is still unclear how SZ risk genes interfere with NMDAR-mediated synaptic transmission in diverse inhibitory interneuron populations. One putative risk gene is neuregulin 1 (*NRG1*), which signals via the receptor tyrosine kinase ErbB4, itself a schizophrenia risk gene. The type I isoform of *NRG1* shows increased expression in the brain of SZ patients, and ErbB4 is enriched in GABAergic interneurons expressing parvalbumin (PV) or cholecystokinin (CCK). Here, we investigated ErbB4 expression and synaptic transmission in interneuronal populations of the hippocampus of transgenic mice overexpressing NRG1 type I (NRG1^tg-type-I^ mice). Immunohistochemical analyses confirmed that ErbB4 was coexpressed with either PV or CCK in hippocampal interneurons, but we observed a reduced number of ErbB4-immunopositive interneurons in the NRG1^tg-type-I^ mice. NMDAR-mediated currents in interneurons expressing PV (including PV^+^ basket cells) or CCK were reduced in NRG1^tg-type-I^ mice compared to their littermate controls. We found no difference in AMPA receptor–mediated currents. Optogenetic activation (5 pulses at 20 Hz) of local glutamatergic fibers revealed a decreased NMDAR-mediated contribution to disynaptic GABAergic inhibition of pyramidal cells in the NRG1^tg-type-I^ mice. GABAergic synaptic transmission from either PV^+^ or CCK^+^ interneurons, and glutamatergic transmission onto pyramidal cells, did not significantly differ between genotypes. The results indicate that synaptic NMDAR-mediated signaling in hippocampal interneurons is sensitive to chronically elevated NGR1 type I levels. This may contribute to the pathophysiological consequences of increased *NRG1* expression in SZ.

## Significance Statement

Hypofunction of NMDA receptors in inhibitory GABAergic interneurons is implicated in pathophysiology of schizophrenia (SZ), but it is largely unknown how SZ risk genes interfere with NMDAR-mediated signaling in specific interneurons. We investigated synaptic transmission in hippocampus of mice overexpressing the type I isoform of the putative SZ risk gene, NRG1, and found markedly reduced NMDAR-mediated synaptic responses in GABAergic interneuron types labeled for PV or CCK, which are known to express the NRG1 receptor ErbB4. The NMDAR hypofunction changed synaptic excitatory drive of interneurons during hippocampal network activity. The observed reductions of NMDAR-mediated transmission in these interneurons may contribute to the hippocampal dysfunction observed with increased NGR1 type I expression and may provide a link to the genetic predisposition to SZ.

## Introduction

Many schizophrenia (SZ) susceptibility genes have been linked to *N*-methyl-d-aspartate receptor (NMDAR) signaling (Harrison and Weinberger, 2005; Hall et al., 2015), consistent with the hypothesis that NMDAR hypofunction contributes to the disease pathophysiology (Olney and Farber, 1995; Coyle, 2012; Gonzalez-Burgos and Lewis, 2012). It has been proposed that NMDAR function could particularly be impaired in hippocampal and neocortical GABAergic interneurons in the disorder, compromising recurrent inhibition (Carlén et al., 2012; Curley and Lewis, 2012; Gilmour et al., 2012). Two prominent GABAergic inhibitory interneuron subpopulations, defined by mutually exclusive markers parvalbumin (PV) or cholecystokinin (CCK), are strongly involved through recurrent inhibition in rhythmic network activities in the neocortex and hippocampus (Cobb et al., 1995; Ellender and Paulsen, 2010; Manseau et al., 2010; Lasztóczi et al., 2011; Buzsáki and Wang, 2012; Fasano et al., 2017; Pelkey et al., 2017). Disrupted function of either of these interneuron populations in animal models results in alterations of coordinated neuronal network activities, particularly the synchronous gamma frequency (20–80 Hz) oscillations, and causes behavioral changes associated with the disorder (Belforte et al., 2010; Nakazawa et al., 2012; Brown et al., 2014; Schmidt et al., 2014; Cho et al., 2015; Gonzalez-Burgos et al., 2015; Schmidt and Mirnics, 2015; Huang et al., 2016; Del Pino et al., 2017; Medrihan et al., 2017; Vargish et al., 2017). However, whether and how specific SZ susceptibility genes interfere with NMDAR-mediated synaptic signaling in these interneurons is still not well known (Gonzalez-Burgos and Lewis, 2012; Vullhorst et al., 2015). In this respect, the gene for neuregulin 1 (*NRG1*) is a relevant candidate to study because diverse evidence links it to NMDAR function and SZ pathogenesis (Stefansson et al., 2002; Corfas et al., 2004; Gu et al., 2005; Hahn et al., 2006; Law et al., 2006; Bjarnadottir et al., 2007; Chong et al., 2008; Pitcher et al., 2011; Weickert et al., 2013). Moreover, the main receptor for NRG1 signaling, ErbB4, itself a schizophrenia risk gene (Schizophrenia Working Group of the Psychiatric Genomics Consortium, 2014), is expressed in PV^+^ and CCK^+^ GABAergic interneurons but not in glutamatergic pyramidal cells (Vullhorst et al., 2009; Fazzari et al., 2010; Neddens et al., 2011; Del Pino et al., 2017).

NRG1 has several functionally distinct isoforms, of which type I (among others) has been reported to be overexpressed in SZ (Hashimoto et al., 2004; Law et al., 2006). Overexpression of NRG1 type I mRNA, or administration of the protein in early postnatal development, results in pathophysiological changes reminiscent of schizophrenia endophenotype in animal models: alterations in rhythmic gamma-frequency network oscillations (Deakin et al., 2012) and synaptic plasticity (Agarwal et al., 2014), and a behavioral phenotype including age-emergent impairment of hippocampal working memory (Chen et al., 2008; Deakin et al., 2009; Kato et al., 2011; Yin et al., 2013; Luo et al., 2014). These findings together suggest that NRG1-ErbB4 signaling may regulate glutamatergic NMDAR-mediated transmission in interneurons, and that alterations in this mechanism might contribute to the pathophysiology of SZ. To investigate this possibility, we have studied synaptic function in hippocampal interneurons expressing PV or CCK in mice overexpressing NRG1 type 1, using a combination of electrophysiological, optogenetic, and immunohistochemical techniques.

## Materials and Methods

### Ethics statement

All animal procedures were performed in accordance with British Home Office regulations and personal and project licenses held by the authors, following local ethics review at the University of Oxford (UK).

### Experimental animals

**Figure 1.**
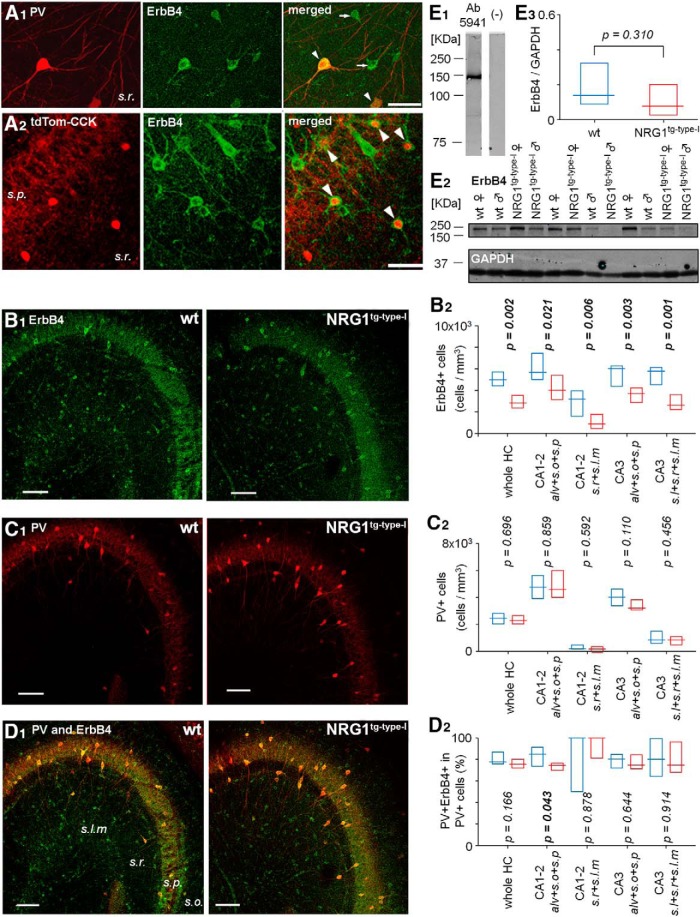
ErbB4 expression in PV^+^ and PV^–^ interneurons and the ErbB4 expression levels in hippocampus of WT and the NRG1^tg-type-I^ mice. ***A***, Immunostaining for ErbB4, the NRG1 receptor, in the ventral hippocampus CA3 area neurons using highly specific rabbit anti-ErbB4 (polyclonal anti-antiserum 5941; Neddens and Buonanno, 2010). ***A1***, Double immunolabeling for PV (Cy3) and ErbB4 (Alexa488). Merged image shows double-labeled neurons (arrowhead) and ErbB4^+^ interneurons immunonegative for PV (arrows). *s.r*, *stratum radiatum.* ***A2***, In mice with genetic fluorescence marker (tdTomato) in CCK cells (tdTom-CCK), ErbB4 immunostaining with Alexa Fluor 488 shows the expression in many CCK^+^ neurons in *s.r.* and *stratum pyramidale* (*s.p.*). Cre-dependent tdTomato signal is strong in putative CA3 interneurons (soma in *s.r*.) and weaker in *s.p.*, where the majority of pyramidal cell somata are located (contrast adjustment in the image). In merged image, arrowheads point at interneuron somata with both fluorescent signals. Scale bars, 50 µm. Confocal microscope images. ***B–D***, Cell density analysis of hippocampal interneurons immunopositive for ErbB4 in the WT and NRG1 type I–overexpressing mice (NRG1^tg-type-I^ mice). ***B1***, ErbB4 immunoreaction (20-µm stack image) in sample hippocampal sections of WT (left) and NRG1^tg-type-I^ mice (right). Scale bar, 100 µm. ***B2***, Box plots show ErbB4^+^ cell soma density (measured up to 20-µm depth from the section surface) in WT (blue, *n* = 9 sections in 3 mice) and NRG1^tg-type-I^ (red, 12 sections in 3 mice) mice hippocampi. The plot shows median and interquartile range. Fewer ErbB4^+^ somata were detected in the NRG1^tg-type-I^ mice compared to the WT mice in all hippocampal areas. From the left: whole hippocampus including areas CA1, CA2, and CA3; area CA1–2 restricted to *alveus*, *stratum oriens*, and *stratum pyramidale*; area CA1–2 restricted to *stratum radiatum* and *lacunosum-moleculare*; area CA3 containing *alveus* with *strata oriens* and *pyramidale*; and area CA3 with *strata lucidum* and *radiatum* and *lacunosum-moleculare*. *p*-values compare data between genotypes (Mann–Whitney *U* test). ***C1***, Immunoreaction for PV in the same sections as in ***B1***. ***C2***, Cell density analyses show no difference in the observed PV^+^ cell somata between the two genotypes as indicated by *p* values (Mann–Whitney *U* test). Box plots as in ***B2***. ***D1***, Merged ErbB4 and PV immunolabeling in the sample sections above. ***D2***, Box plots show proportion of the double-labeled cells (co-immunoreactive for ErbB4 and PV) in the PV^+^ cell population in WT and NRG1^tg-type-I^ mice. The analyses show unaltered proportion in the whole hippocampus and in most subregions compared separately. The significant *p* value is bolded. ***E***, Immunoblot analysis of ErbB4 expression levels in WT and NRG1^tg-type-I^ mice using hippocampal extracts. ***E1***, The antibody against ErbB4 detects a band of the predicted protein size (∼150 kDa) in hippocampal protein extracts. Left lane, no nonspecific bands were detected in the secondary-only antibody control (right lane). ***E2***, Hippocampal extracts from 6 WT and 6 NRG1^tg-type-I^ mice of both genders (3 males and 3 females in each genotype in scrambled order) tested for ErbB4 expression. GAPDH was used as a loading control. ***E3***, Box plot shows (mean and interquartile range) densitometry analysis comparison of the ErbB4 levels normalized by the GAPDH in the 12 hippocampal extracts (6 in both genotypes including 3 males and 3 females). The results indicate a general trend to lower ErbB4 levels in NRG1^tg-type-I^ mice, but with no significant difference between the genotypes (Mann–Whitney *U* test).

**Figure 2.**
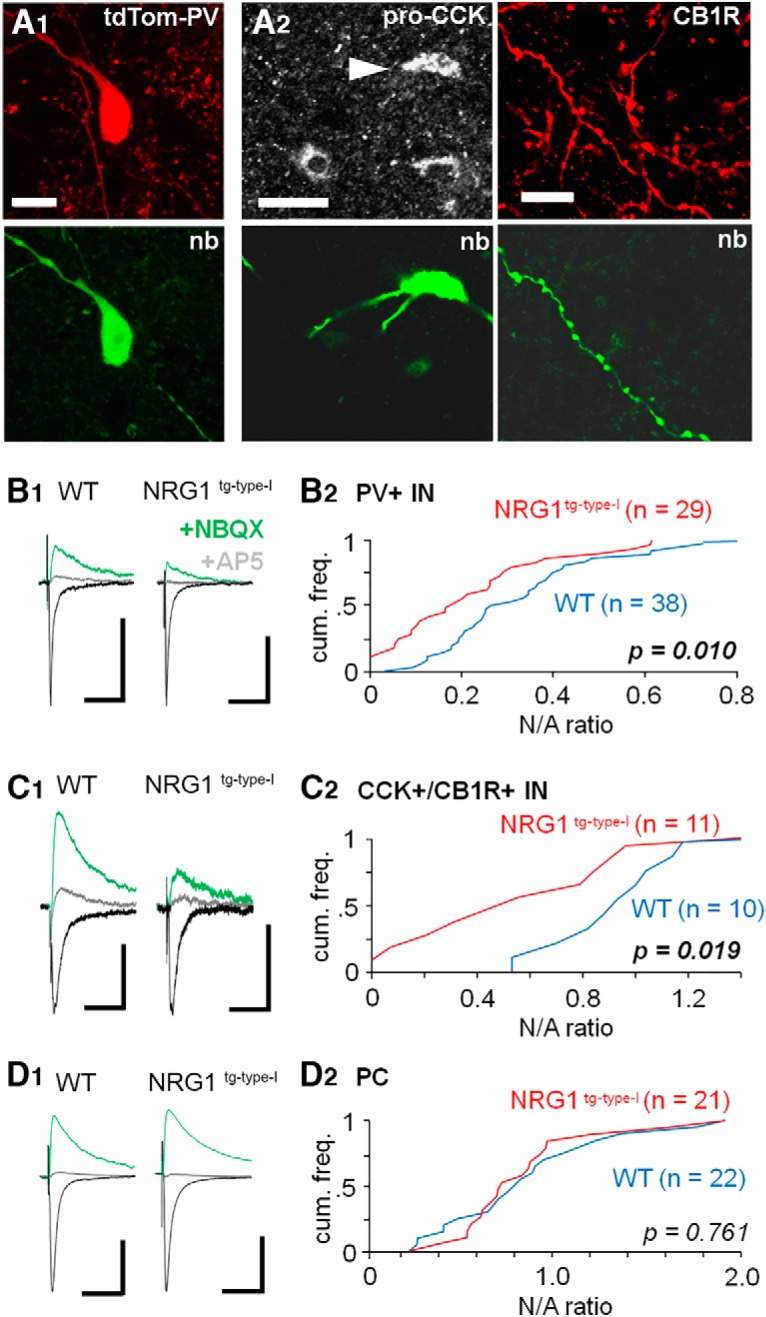
Reduced synaptic NMDAR-mediated currents in hippocampal interneurons expressing PV or CCK in the NRG1^tg-type-I^ mice. ***A***, Interneurons expressing PV or CCK in the CA3 area. ***A1***, Sample image of a recorded PV interneuron identified by PV expression-dependent fluorescent genetic marker tdTomato (tdTom-PV). Recorded cells were also visualized with filled neurobiotin (nb, Alexa Fluor 488). ***A2***, Recorded cells not showing tdTomato signal were identified as CCK^+^ interneurons *post hoc* with positive somatic immunoreaction for pro-CCK (left; Cy5, arrowhead) or in the absence of recovered soma and dendrites (right) by positive reaction for axonal cannabinoid receptor type 1 (CB1R, Cy3). Scale bars from left: 10, 20, and 10 µm, respectively. ***B***, Reduced NMDAR- versus AMPAR-mediated EPSCs ratio (N/A ratio) in glutamatergic synaptic input to interneurons expressing PV. Electrical stimulation was applied in CA3 *stratum oriens* aiming to activate associative/commissural pathways. AMPAR-mediated EPSCs were recorded at –60 mV (in PiTX, 100 µm) and blocked by NBQX (25 µm) to record NMDAR-mediated EPSCs (at 40 mV from their reversal potential). ***B1***, Averaged EPSCs (10 traces) in sample PV^+^ interneurons in WT and NRG1^tg-type-I^ mouse (black, AMPAR EPSCs*;* green, NMDAR EPSCs in the presence of NBQX*;* gray, following application of NMDAR blocker DL-AP5). Scale bars, 100 pA, 25 ms. ***B2***, Cumulative histograms of the N/A amplitude ratios in all studied PV^+^ interneurons (WT, blue line; NRG1^tg-type-I^, red line). *p* indicates difference between the genotypes (Mann–Whitney *U* test). ***C***, Reduced N/A ratio in glutamatergic synaptic input to the CCK^+^ interneurons. ***C1***, Averaged EPSCs (10) in sample cells in the WT and in the NRG1^tg-type-I^ mouse with scaling as above. ***C2***, Cumulative histogram quantifying the N/A ratios in CCK^+^ interneurons with *p* indicating significant difference between the genotypes (Mann–Whitney *U* test). ***D***, The N/A ratio is unaltered between the genotypes in the CA3 pyramidal cells. ***D1***, Averaged EPSCs (10 traces) in sample pyramidal cells with scaling as above. ***D2***, Cumulative histograms of the N/A ratios.

**Figure 3.**
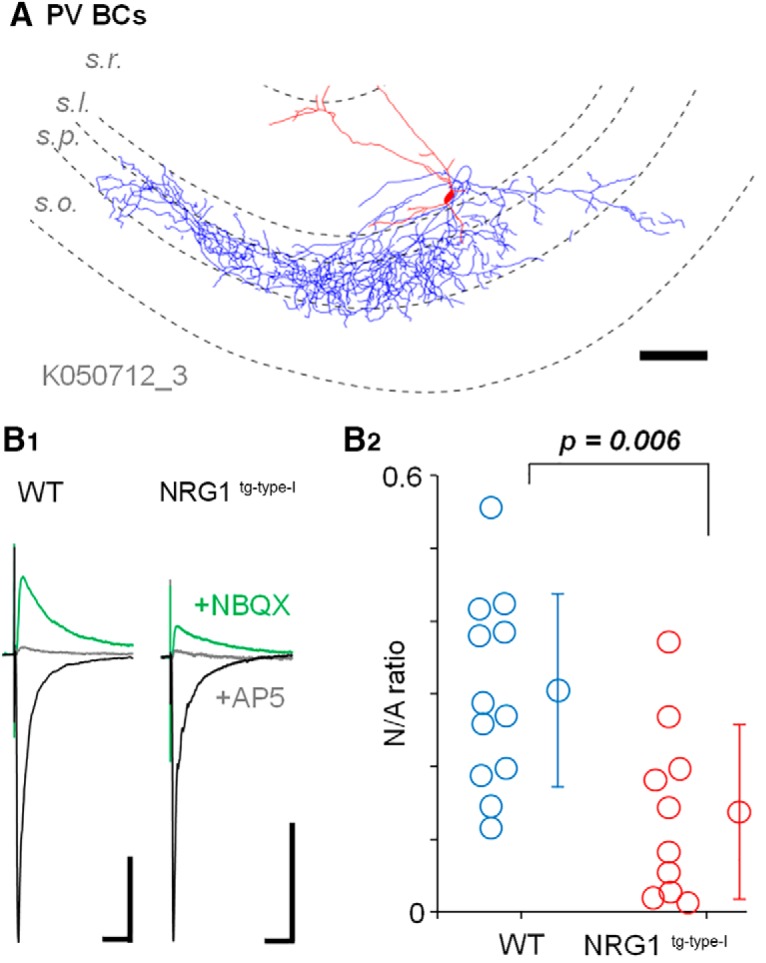
Reduced synaptic NMDAR-mediated currents in identified PV basket cells in the NRG1^tg-type-I^ mice. Identified PV basket cells (PVBCs) in the recorded interneuron population (see Fig. 2) show reduced N/A ratio in the NRG1^tg-type-I^ mice. ***A***, Illustration of a sample PVBC (70-μm-thick section) in WT (axon, blue; soma and dendrites, red; s.r., *stratum radiatum*; s.luc., *lucidum*; s.*p*., *pyramidale*; s.o., *oriens*). Scale, 100 μm. ***B***, The N/A EPSC amplitude ratio in identified basket cells. ***B1***, Averaged (10) EPSCs in a PVBC from WT and NRG1^tg-type-I^ mouse. Black, AMPAR EPSCs at –60 mV*;* green, EPSCs (at 40 mV from their reversal potential in the presence of NBQX, 25 μm); gray, EPSCs following the application of DL-AP5 (100 μm). PiTX (100 µm) was present in all experiments. Scale bars, 50 pA, 25 ms. ***B2***, Plot shows N/A ratio of every identified PVBC in WT (blue circles) and NRG1^tg-type-I^ mice (red circles), and their mean ± SEM (*n* = 10 and 10 cells). *p* value indicates highly significant difference (*t* test).

**Figure 4.**
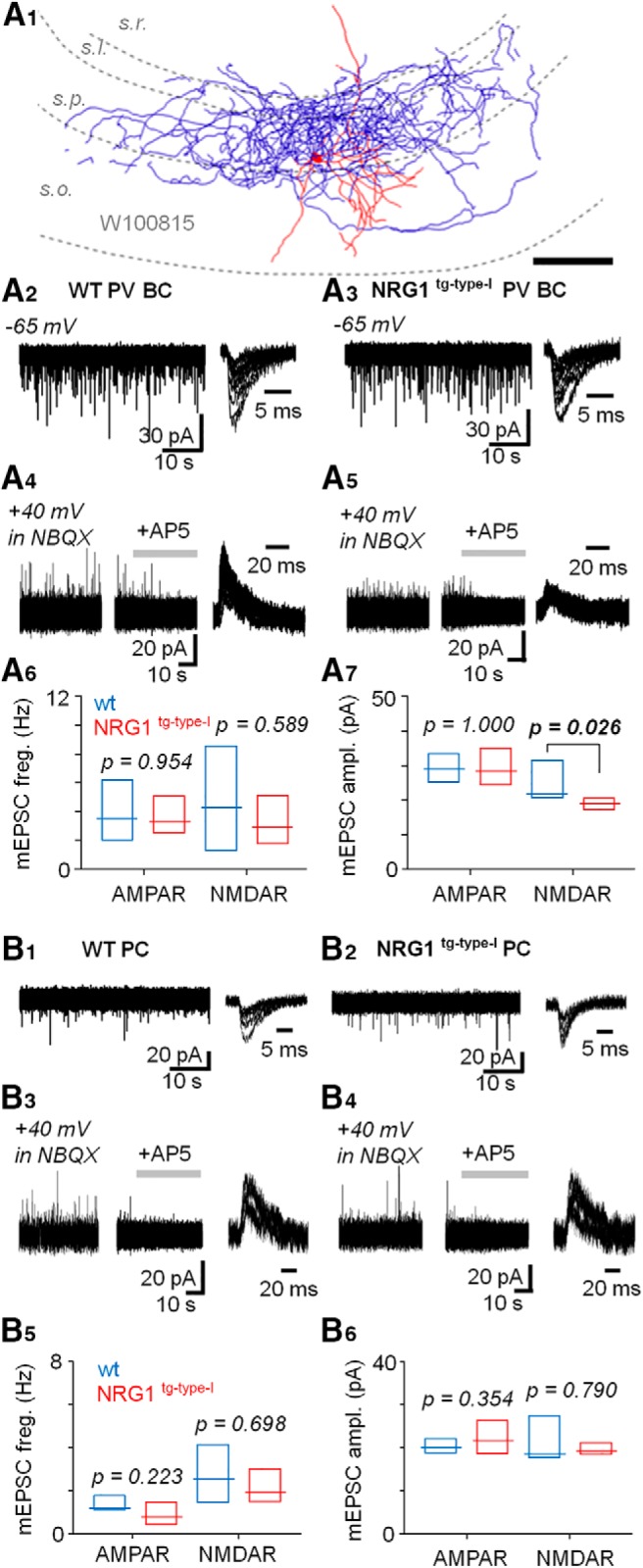
Quantal current analysis in parvalbumin basket cells shows unaltered AMPAR mEPSCs in the NRG1^tg-type-I^ mice. ***A***, Recording of miniature AMPAR- and NMDAR-mediated EPSCs (mEPSCs) in identified CA3 area PVBCs (in the presence of TTX 1 μm and PiTX 100 μm). ***A1***, Illustration of a recorded and partially reconstructed PVBC (70-μm-thick section) in WT mouse. Scale, 100 μm. ***A2***, AMPAR mEPSCs in a PVBC in the WT mouse (at –65 mV). Left, the mEPSCs shown in 45-s time window. Right, six events superimposed in 15-ms time window. ***A3***, AMPAR mEPSCs in a PVBC in the NRG1^tg-type-I^ mouse. ***A4***, NMDAR mEPSCs in the same WT mouse PVBC as in ***A2*** after blockade of AMPARs by NBQX (25 µm, recorded at 40 mV). Left, the mEPSCs shown in 45-s time window. Middle, the mEPSCs blockade with DL-AP5 (100 µm, application indicated by horizontal bar). Right, six superimposed mEPSCs in 80-ms time window. ***A5***, NMDAR mEPSCs blocked by DL-AP5 in NRG1^tg-type-I^ mouse PVBC shown in ***A3***. ***A6***, Box plot (median, interquartile range) summarizes AMPAR and NMDAR mEPSC frequency (measured at least 3 min) in PVBC in WT (blue) and NRG1^tg-type-I^ (red) mice. ***A7***, Box plot summarizes mEPSC amplitude. Note moderately but significantly smaller NMDAR mEPSC in the NRG1^tg-type-I^ mice PVBCs (Mann–Whitney *U* test). The significant *p* value is bolded. ***B***, Unaltered NMDAR- and AMPAR-mediated mEPSCs in the CA3 area pyramidal cells in NRG1^tg-type-I^ mice. ***B1***, ***B2***, Sample traces showing AMPAR mEPSCs in pyramidal cells of both genotypes. ***B3***, ***B4***, Respectively, NMDAR EPSCs in the same cells. ***B5***, Box plot (median, interquartile range) summarizing the AMPAR- and NMDAR-mediated mEPSC frequency (WT, blue, NRG1^tg-type-I^, red). ***B6***, Summary of he AMPAR and NMDAR mEPSC amplitudes in the two genotypes (Mann–Whitney *U* test).

Experiments were conducted on heterozygous (at least 1 month old) NRG1 type I transgenic (NRG1^tg-type-I^) mice of either sex, overexpressing NRG1 type I (β1a-isoform) under a Thy-1.2 promoter (RRID:MGI:3530784; Michailov et al., 2004). To specifically express fluorescent marker in PV interneurons, PV-Cre^+/+^ mice (The Jackson Laboratory, B6;129P2-Pvalbtm1[cre]Arbr/J; RRID:IMSR_JAX:017320) were crossbred with Ai9^+/+^ mice (The Jackson Laboratory, B6.Cg-Gt[ROSA]26Sortm9[CAG-tdTomato]Hze/J; RRID:IMSR_JAX:007909) to produce tdTomato expression in the PV^+^ cells (Figs. 1, 2 and 3). The female offspring were further crossed with the NRG1^tg-type-I +/−^ males. For the experiments in Fig. 4, Lhx6-eGFP+/− females expressing GFP in PV cells (The Jackson Laboratory, Tg[Lhx6-EGFP]BP221Gsat/M, RRID:MMRRC_000246-MU) were crossbred with male NRG1^tg-type-I +/−^ mice, and anatomically identified basket cells in hippocampal slices (Nissen et al., 2010) were defined as PV^+^ basket cells (PVBCs) and confirmed immunonegative for axonal cannabinoid receptor type 1 (CB1R; Armstrong and Soltesz, 2012). To express fluorescent marker in CCK neurons, BAC-CCK-Cre^+/−^ mice (Geibel et al., 2014; RRID:MGI:5575864) were crossed with the Ai9^+/+^ mice for tdTomato expression in the CCK^+^ cells. For the virus transduction studies, PV-Cre^+/+^ females (The Jackson Laboratory, B6;129P2-Pvalbtm1[cre]Arbr/J; RRID:IMSR_JAX:017320), heterozygous BAC-CCK- Cre +/− females (RRID:MGI:5575864), or CaMKII-Cre^+/+^ females (B6.Cg-Tg[Camk2a-cre]T29-1Stl/J; RRID:IMSR_JAX:005359) were crossbred with male NRG1^tg-type-I +/−^ mice. The Cre-expressing NRG1^tg-type-I^ and control littermates were injected with adeno-associated virus construct encoding opsin.

### Opsin construct transduction

Mice were anesthetized with 2%–4% isoflurane (CHEBI: 6015). AAV2-ChR2-eYFP (in some cases AAV5-ChR2-eYFP) was stereotactically injected via 33-gauge needle attached to a Microlitre Syringe (Hamilton) into midventral CA3 or dorsal CA1 hippocampus. The vector sequence was: pAAV-EF1a-sCreDIO hChR2(H134R)-EYFP-WPRE (Vector Core Services, Gene Therapy Center Virus, University of North Carolina). In each hemisphere, a craniotomy was performed using a micro-torque, and a total volume of 800 nl virus suspension (viral particle suspension titer 4 × 10^12^/mL) was delivered at 80 nl/min by a Micro Syringe Pump Controller (World Precision Instruments). The scalp incision was sutured, and mice were allowed to recover for 10–21 d. Light exposure of brain tissue during preparation of slices was minimized to avoid photoactivation of ChR2. In experiments, ChR2 was activated by a fixed-spot laser (Laser nominal maximum power 100 mW; Rapp OptoElectronics) light (20-µm diameter to evoke IPSCs with minimal stimulation of GABAergic fibers, and 80-µm diameter in experiments stimulating glutamatergic fibers with 20-Hz train stimulation) via the microscope objective.

### Identification of interneuron populations and pyramidal cells

CCK interneurons in Fig. 1 were tagged by the fluorescent marker tdTomato using the crossed mouse line: BAC-CCK-Cre tg with Ai9 mice. In Fig. 2, CCK-expressing interneurons were identified with positive immunoreaction for somatic pro-CCK or by positive immunoreaction for axonal CB1R when the soma recovery was compromised. In Figs. 1, 2, and 3, the PV-expressing cells were identified by genetic fluorescence marker in PV-Cre mice crossed with Ai9 mice. Recorded cells were filled with neurobiotin (0.3% w/v) and visualized, and some were anatomically identified as basket cells by their characteristic predominant axon distribution in *str. pyramidale* and the lack of axo-axonic cell axon terminal cartridges (Klausberger and Somogyi, 2008). In addition, the basket cells in Fig. 4 were confirmed immunonegative for axonal CB1R (Katona et al., 1999; Tsou et al., 1999; Bodor et al., 2005; Armstrong and Soltesz, 2012). Pyramidal cells (PCs) were identified by their somatodendritic structure with mushroom spines along the dendrites.

### Electrophysiological recordings

Mice were anesthetized with sodium-pentobarbitone and decapitated. After brain removal, horizontal (for mid-ventral hippocampus) or coronal (for dorsal hippocampus) brain slices (250 µm) were cut using a vibrating microtome (Microm HM650V) in oxygenated (95% O_2_/5% CO_2_) ice-cold (0–4°C) cutting solution. The composition of the cutting solution was (in mm): 75 sucrose, 87 NaCl, 2.5 KCl, 0.5 CaCl_2_, 7 MgCl_2_, 1.0 NaH_2_PO_4_, 25 NaHCO_3_, 25 glucose, pH 7.4, bubbled with 95% O_2_/5% CO_2_. Slices were kept submerged at 32°C in the sucrose solution for 20–25 min before being transferred to an interface chamber in which they were maintained in Earle’s balanced salt solution (Thermo Fisher Scientific, 14155063) with 3 mm Mg^2+^ and 1 mm Ca^2+^ at room temperature (20–24°C) for at least 60 min before starting experiments. In the experiments, the slices were superfused with oxygenated recording solution at 5 mL/min in a submerged-type recording chamber at 30°C (Luigs & Neumann) mounted on Olympus BX51 microscope stage (20× objective, 2–4 zoom) with epifluorescence and filters (eGFP, eYFP, tdTomato) and DIC-IR with a CCD camera (Till Photonics). The superfusion solution was (in mm): 119 NaCl, 2.5 KCl, 2.5 CaCl_2_, 1.3 MgSO_4_, 1.25 NaH_2_PO_4_, 25 NaHCO_3_, and 11 glucose, final pH 7.4 (equilibrated with 95% O_2_/5% CO_2_).

**Figure 5.**
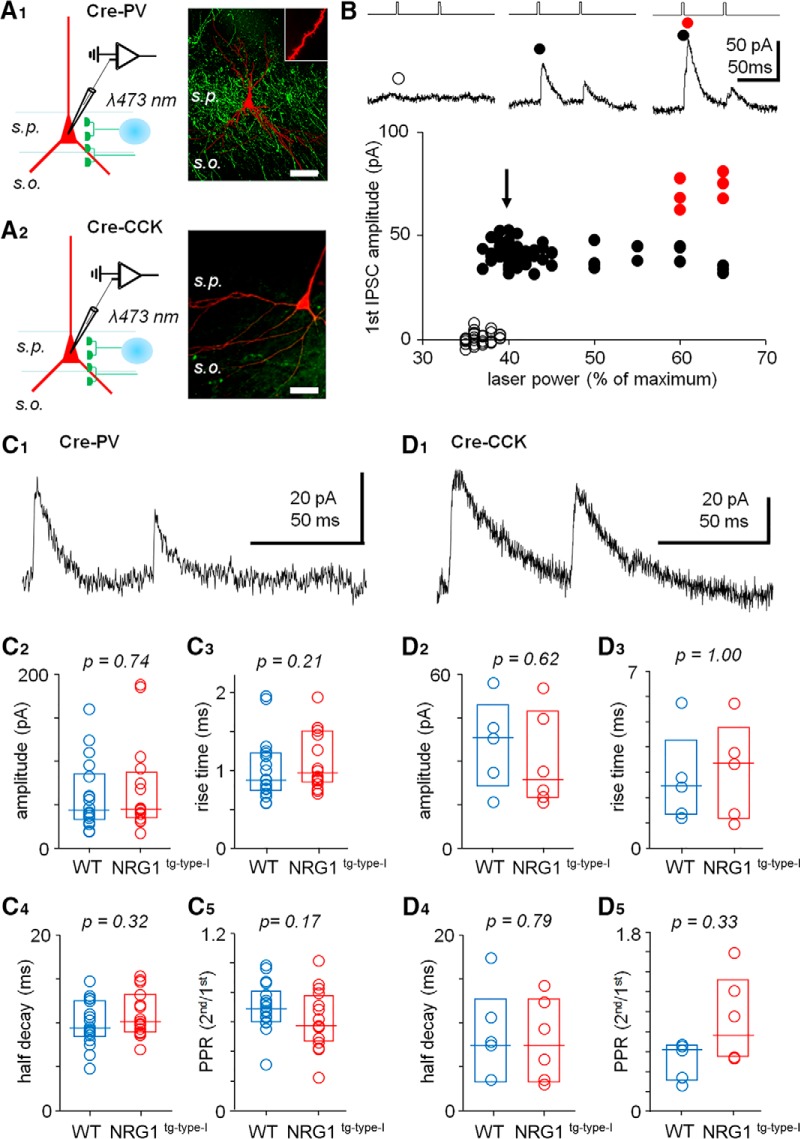
GABAergic synaptic transmission from either PV^+^ or CCK^+^ cells is not significantly altered in NRG1^tg-type-I^ mice. ***A***, Experimental design showing optogenetic stimulation (at 473-nm laser spot, 20-μm diameter) of GABAergic fibers in the CA3 *stratum pyramidale* in slices from ChR2-eYFP–transfected mice expressing Cre-protein in either PV^+^ cells (***A1***) or CCK^+^ cells (***A2***). Left, schematic illustration of the experiment with whole-cell recording in CA3 PCs and optogenetic stimulation focused on *stratum pyramidale (s.p.)*. Right, confocal microscope images from sample slices (visualized *post hoc*) showing eYFP fluorescence (green) in the PV- (above) or CCK-Cre mice. Postsynaptic neurobiotin-filled pyramidal cells are shown red with an inset of a spiny pyramidal cell apical dendrite. Scale, 50 μm. ***B***, Sample experiment showing optogenetically evoked GABAergic IPSCs in a postsynaptic pyramidal cell using minimal stimulation. Monosynaptic IPSCs (black circles) were evoked by smallest stimulation power eliciting IPSCs in the PC. Open circles, failures; red circles, additional IPSCs elicited by increased stimulation power. Timing of laser pulses with representative IPSCs in the experiment is shown above. ***C***, The optogenetically evoked GABAergic IPSCs from PV cell fibers do not differ significantly between WT mice (blue) and NRG1^tg-type-I^ mice (red; Mann–Whitney *U* test). ***C1***, A sample trace. Box plots (median, interquartile range) show data from all the PCs studied. ***C2***, The evoked IPSC amplitudes. ***C3***, The IPSC half decay. ***C4***, The IPSC rise time. ***C5***, The IPSC paired-pulse (50 ms) ratio (2nd/1st IPSC amplitude). *p* values with Mann-Whitney test. ***D***, The IPSCs from CCK-fibers do not show significant difference between the genotypes. ***D1***, Sample trace. ***D2–D5***, The IPSC amplitude, IPSC half decay, rise time, and paired-pulse ratio, respectively (Mann–Whitney test).

**Figure 6.**
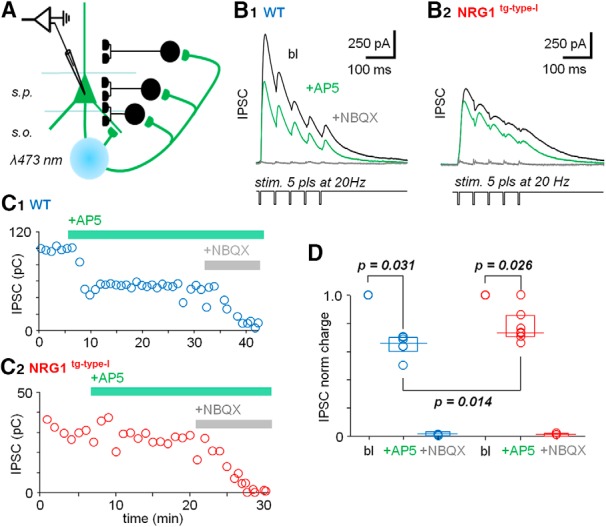
Reduced NMDAR-driven recurrent hippocampal inhibition in NRG1^tg-type-I^ mice. ***A***, Schematic summarizes the experimental design. Optogenetic stimulation of CA1 area pyramidal cell fibers expressing ChR2 (green, CAMKII-Cre mice transfected with AAV2-ChR2-eYFP). Recurrent inhibitory IPSCs are generated by laser spot (473-nm, 3-ms) stimulation focused in *stratum pyramidale* (*s.p.*) and *oriens* (*s.o.*). ***B***, Sample experiments showing averaged (5) recurrent IPSCs in the CA1 pyramidal cells, evoked by the optogenetic stimulation (5 pulses at 20 Hz) in WT (***B1***) and NRG1^tg-type-I^ (***B2***) mice. Black traces show IPSCs in baseline; green is in the presence of NMDAR blocker DL-AP5 (100 μm, at 5–8 min after DL-AP5 application). The IPSCs were recorded at the reversal potential of EPSCs. The IPSCs were fully blocked with NBQX (25 µm, gray traces). ***C***, Plots show the recurrent IPSC charge in sample experiments in the WT (***C1***) and NRG1^tg-type-I^ (***C2***) mouse. Wash-in of DL-AP5 and NBQX is indicated by green and gray horizontal bars, respectively. ***D***, The hippocampal recurrent IPSCs in the NRG1^tg-type-I^ mice show reduced sensitivity to the NMDAR antagonist. Box plot (median, interquartile range) summarizes the effect of DL-AP5 (100 µm) on the recurrent IPSC charge in WT (blue) and NRG1^tg-type-I^ (red) mice. The IPSC charge in the presence of DL-AP5 (and in the presence of NBQX) is normalized with the baseline for each experiment. *p* value with Mann–Whitney *U* test.

Borosilicate-glass microelectrodes were pulled (P-97, Sutter Instrument) from GC150F-10 capillaries (Harvard Apparatus). Pipettes (6–8 MΩ) were filled (Figs. 2, 3, and 4) with (in mm): 145 Cs-methanesulfonate, 20 HEPES, 10 CsOH, 8 NaCl, 0.2 CsOH-EGTA, 2 ATP-Mg, 0.3 GTP-Na, 5 QX-314, and 0.2%–0.5% neurobiotin (295 mOsm, pH 7.2). In Figs. 5 and 6, 145 K-gluconate or K-methanesulfonate (with 10 KOH, and 0.2 K-EGTA) were used instead. Recordings with >30% change in access resistance were excluded. Liquid-junction potential was not corrected. Data were recorded with a Multiclamp 700B amplifier, low-pass filtered (cutoff frequency ≥2 kHz), digitized (≥10 kHz, Digidata 1400), acquired by Clampex, and analyzed by pClamp10.2 (Molecular Devices, SCR_011323).

Extracellular electrical stimuli were applied via a bipolar electrode (50–100 µs, 50–400 µA) in *stratum oriens* and current isolator (CBAPC75PL1, FHC) every 15 s. Synaptic currents were *post hoc* lowpass filtered at 1 KHz. Pharmacologically isolated AMPA receptor (AMPAR)-mediated EPSC peak amplitude was recorded at –60 mV, and the NMDAR-mediated EPSC amplitude was measured in the presence of the AMPA/kainate receptor blocker NBQX at a membrane potential 40 mV positive to their measured reversal potential estimated by a linear fitting curve of the current–voltage relation for at least 20 evoked NMDAR EPSCs measured between –20 and 65 mV (Deleuze and Huguenard, 2016). In cells where no NMDAR EPSC was detected, the current was defined as 0.mEPSC recordings (2 min for AMPAR and 2 min for NMDAR mEPSCs) were acquired at 20 kHz and bandpass filtered offline (cutoff frequencies 4 Hz to 5 or 6 kHz at –65 mV, 2–500 Hz at 40 mV) for analysis. Events were detected with an amplitude threshold-crossing algorithm in pClamp (Molecular Devices, SCR_011323). Criteria for threshold detection for NMDAR mEPSCs (at 40 mV) were amplitude threshold 7 pA, duration 0.8–200 ms, with noise rejection 0.8 ms. For the AMPAR mEPSCs (at –65 mV) the amplitude threshold was 5 pA, duration 0.5–100 ms, with noise rejection 0.5 ms) evaluated after blockade of AMPARs with NBQX (25 μm). The same detection criteria were employed for all cells. Number of AMPAR mEPSCs investigated in the analyses were as follows: in wild-type (WT) basket cells (median and interquartile range), 424 and 279–680 events (7 cells); in NRG1^tg-type-I^ basket cells, 394 and 301–470 events (6 cells); in WT pyramidal cells, 134 and 128–205 events (7 cells); in NRG1^tg-type-I^ pyramidal cells, 95 and 64–150 events (10 cells). The numbers of NMDAR mEPSCs measured in similar time window were as follows: in WT basket cells (median and interquartile range), 513 and 178–792 events (6 cells); in NRG1^tg-type-I^ basket cells, 348 and 280–520 events (6 cells). mEPSC frequency was calculated from the 2-min time window as the event occurrence in Hz. Average mEPSC amplitude was calculated in each cell from all events occurring in the 2-min time window.

In experiments using optogenetic stimulation of GABAergic fibers, the monosynaptic IPSCs were measured at 0 to –10 mV. Optogenetic stimulation of the glutamatergic fibers (5 pulses at 20 Hz) was applied every 30 s while the disynaptic IPSCs were recorded (on average at 11 mV; see Results) in postsynaptic pyramidal cells. The optogenetically evoked postsynaptic currents were lowpass filtered offline at 1 kHz, and the evoked postsynaptic current charge was analyzed with pClamp10.2 (Molecular Devices, SCR_011323).

### Drugs

Drugs were purchased as follows: 2,3-dioxo-6-nitro-1,2,3,4-tetrahydrobenzo[f]quinoxaline-7-sulfonamide disodium salt (NBQX) from Abcam; dl-2-amino-5-phosphonopentanoic acid sodium salt (DL-AP5), picrotoxin (PiTX), CGP55845, 4-[(2*S*)-2-[(5-isoquinolinylsulfonyl)methylamino]-3-oxo-3-(4-phenyl-1-piperazinyl)propyl] phenyl isoquinolinesulfonic acid ester (KN-62), and (*RS*)-α-methyl-4-carboxyphenylglycine (MCPG) from Tocris Bioscience. Stocks were diluted (1:1000) in ddH_2_O, DMSO, or ethanol.

### Statistics

A *t* test was used for data that were normally distributed (Shapiro–Wilk test) and with *n* ≥ 10 in tested groups. Otherwise, a Mann–Whitney *U* test or rank sum test was used.

### Anatomic and immunohistochemical processes

After whole-cell recordings, slices were fixed overnight at 4°C in 4% paraformaldehyde (NIFCHEM:birnlex 3072_2), 0.05% glutaraldehyde (NIFINV:birnlex 3070_2), and 0.2% picric acid in 0.1 m sodium phosphate buffer (PB), then washed in 0.1 m PB. Slices were embedded in 20% gelatin and sectioned (40–60 µm) with a microtome (Leica VT1000) in 0.1 m PB, then washed in 50 mm Tris-buffered saline (TBS, pH 7.4) with 0.3% Triton X-100 (TBS-Tx) and incubated with streptavidin-Alexa Fluor 488 (1:2000, Invitrogen, S-32354) or -Cy3 (1:2000, Thermo Fisher, S-A1010), and finally washed in 50 mm TBS-Tx. Sections mounted in Vectashield (Vector Laboratories) were examined with an epifluorescence microscope (DM5000-B, Leica Microsystems) using appropriate filter sets and a CCD camera (ORCA-ER, Hamamatsu).

Sections for immunoreactions were washed in 50 mm TBS-Tx, blocked in 20% normal horse serum (NHS, Vector Laboratories) in TBS-Tx for at least 1 h at room temperature (20–24°C), and incubated in primary antibodies for 48 h at 4°C in TBS-Tx with 1% NHS. Fluorochrome-conjugated secondary antibodies were applied overnight at 4°C in TBS-Tx with 1% NHS. Mounted sections in Vectashield were evaluated at ≥40× magnification using confocal laser-scanning microscopy (LSM710, Carl Zeiss) with Zen2008 software. Digital micrographs were constructed from *z*-stacks with ImageJ software (SCR:003070). Micrographs were only adjusted for brightness and contrast. The primary antibodies used were rabbit anti-ErbB4 (polyclonal anti-antiserum 5941, 1:500; Vullhorst et al., 2009), guinea pig anti-PV (Synaptic Systems, 195004, RRID:AB_2156476, 1:2000), rabbit anti-proCCK (RRID:AB_2571674, 1:500; Morino et al., 1994), guinea pig anti-CB1R (Frontier Science, CB1-GP-af530-1, RRID:AB_2571593, 1:1000). The secondary antibodies were CY5 donkey anti–guinea pig (1:250, Jackson ImmunoResearch, 706-175-148), CY3 donkey anti–guinea pig (1:400, Jackson ImmunoResearch, 706-165-148), Alexa Fluor 647 donkey anti–guinea pig (1:250, Invitrogen, 706-605-148), Alexa Fluor 488 donkey anti-rabbit (1:500, Invitrogen, A21206), Dylight 649 donkey anti-rabbit (1:250, Jackson ImmunoResearch, 711-495-152).

### Cell density analyses

Mice were anesthetized with 2%–4% isoflurane (CHEBI:6015) at a rate of 1.0–1.5 ml/min and then further anesthetized with an intraperitoneal injection of pentobarbitone sodium (20% w/v, dosage 0.2 mg/g; Pharmasol). Animals were perfused with 0.1 m PBS solution (pH 7.4, at 22–24°C) followed by ice-cold fixative solution; 4% w/v paraformaldehyde (NIFCHEM:birnlex 3072_2) with 15% v/v saturated picric acid solution in 0.1 m PB. A vibratome (VT1000S Leica Microsystems) was used for cutting coronal brain sections (60-µm thickness). Sections containing the hippocampal formation were washed (3–5 times, 10 min) with TBS-Tx and blocked with 20% NHS in TBS-Tx for 1 h at room temperature (20–24°C). This was followed by a 2-night incubation with the primary antibodies: rabbit anti-ErbB4 (polyclonal anti-antiserum 5941, 1:500) and guinea pig anti-PV (Synaptic Systems, 195004, RRID:AB2156476, 1:2000) in TBS-Tx with 1% NHS at 4°C. After washes (3–5 times, 10 min each) with TBS-Tx, sections were incubated overnight with Alexa Fluor 488–conjugated and Alexa Fluor 647–conjugated secondary antibodies both raised in donkey, respectively, in TBS-Tx with 1% NHS. Sections containing mid-ventral hippocampus from both hemispheres were scanned using an epifluorescence microscope (AxioImager M2; Zeiss) equipped with Stereoinvestigator software (MBF Bioscience). Optical sections of 1 μm were acquired using a 20× objective at a final depth of 20 μm from the section surface, while the first 1 μm from the section surface was defined as a guard zone and not scanned (Bocchio et al., 2015). Brightness and contrast acquiring settings were adjusted for each section, to achieve good visualization of all positive cells for a specific neuromarker across all section areas. Cell counting was performed offline. Distinct hippocampal regions were visually delineated and analyzed as individual anatomically defined subregions as follows: CA1–2 alveus (alv)/stratum oriens (s.o)/stratum pyramidale (s.p), CA1–2 stratum radiatum (s.r)/stratum lacunosum-moleculare (s.l-m), CA3 alv/s.o/s.p, and CA3 stratum lucidum (s.l)/s.r/s.l-m. Cells were counted when the cell somata or nuclei came into focus with the optical dissector.

### Immunoblotting

Tissue sample homogenates were prepared from mouse hippocampus in ice-cold lysis buffer containing 20 mm Tris (pH 7.5), 50 mm NaCl, 1 mm EDTA, 0.1% SDS, 1% Triton X-100, 2% protease inhibitors (Roche), 1% phosphatase inhibitors Cocktail 2 and 3 (Sigma), using a plastic homogenizer, repeated passages through a syringe, followed by 5-min sonication and 75-min rotation at 4°C. Next, the homogenates were centrifuged at 4000 × *g*, and the supernatant was collected. Lysates were quantified for their total protein content with standard Bradford assay (Bio-Rad), diluted to sample buffer containing 100 mm (or 2× increased) DTT, 10% glycerol, 2% SDS, 2 mm Tris HCl, and 0.1% (w/v) bromophenol blue crystals, and incubated at 95°C for 5 min to denature proteins. Protein lysates were size separated by SDS-PAGE, using 6% or 10% acrylamide gels, and electrophoretically transferred onto nitrocellulose membranes. After blocking in the Odyssey proprietary blocking buffer (LI-COR Biosciences) for 1 h at room temperature (20–24°C), membranes were incubated with the primary antibodies overnight at 4°C (rabbit anti-ErbB4, polyclonal anti-antiserum 5941, 1:900). Rabbit anti-GADPH (1:10,000) in Odyssey blocking buffer was supplemented with 0.01% Tween 20. After washes with PBS × Tx (5 times, 5 min), membranes were incubated with the appropriate fluorescent secondary antibody (goat anti-rabbit IRDYe 800CW, LI-COR Biosciences) for 1 h at 20–24°C. Finally, after five 5-min washes with PBS × Tx, the membranes were scanned with an infrared scanner (Odyssey Clx scanner, LI-COR Biosciences, SCR:014579) and the digital scans were analyzed with Image Studio Lite software (LI-COR Biosciences, SCR:014211).

## Results

### Expression of ErbB4 in the hippocampus of WT and NRG1 type I–overexpressing mice

Given the well-established role of ErbB4 as the major receptor to elicit NRG1 signaling cascades in the brain (Flames et al., 2004; López-Bendito et al., 2006; Krivosheya et al., 2008; Fazzari et al., 2010; Li et al., 2012b), we visualized the ErbB4 receptor in hippocampal interneurons using rabbit anti-ErbB4 (polyclonal anti-antiserum 5941) immunostaining, which shows high epitope specificity (Vullhorst et al., 2009). We found that ErbB4 coexpressed with PV and CCK, the mutually exclusive neuronal markers (Fig. 1*A1*,*A2* ) that label perisoma-targeting (and also some dendrite-targeting) hippocampal interneuron types. Given that NRG1-ErbB4 signaling is known to regulate interneuron migration, survival, and proliferation during neurodevelopment (Flames et al., 2004; Li et al., 2012b), we first investigated whether the NRG1 type I–overexpressing mice showed an altered distribution of ErbB4^+^ interneurons in the hippocampus. Fluorescence imaging-based ErbB4^+^ cell soma counting showed a reduced density in the NRG1^tg-type-I^ mice compared to the WT mice in all subfields (Fig.1*B1*,*B2*). In the whole hippocampus (including the CA1–CA3 areas) of the WT mice, the ErbB4^+^ soma density was 4.98 × 10^3^ cells/mm^3^ (median, interquartile range 4.65–5.66 × 10^3^ cells/mm^3^), and in the NRG1^tg-type-I^ mice, 2.82 × 10^3^ cells/mm^3^ (median, interquartile range 2.37–3.48 × 10^3^ cells/mm^3^; *p* = 0.002, Mann–Whitney *U* test; Fig. 1*B2*). Hippocampal subregions, compared separately, were defined as follows: (1) *stratum pyramidale* with infrapyramidal laminae in the CA1–2 area, (2) suprapyramidal layers in the CA1–2 area, (3) *stratum pyramidale* with infrapyramidal laminae in the CA3 area, (4) suprapyramidal layers in the CA3 area. The subregion-specific soma counting results are illustrated in Fig. 1*B2* . The cell counts in the CA1 and CA2 areas were pooled together because of small size of the CA2, and the result mainly represents the CA1 area.

In contrast, analysis of PV-immunopositive cell somata showed no difference between the two genotypes, in line with a previous study using the same NRG1^tg-type-I^ mouse line (Deakin et al., 2012). Fluorescence imaging-based PV^+^ cell soma counting (Fig. 1*C1*) in the WT mice revealed 2.44 × 10^3^ cells/mm^3^ (median, interquartile range 2.03–2.74 × 10^3^ cells/mm^3^, *n* = 9 sections in 3 mice; Fig. 1*C2*). Correspondingly, the PV^+^ cell soma density analysis in the NRG1^tg-type-I^ mice showed 2.28 × 10^3^ cells/mm^3^ (median, interquartile range 2.03–2.63 × 10^3^ cells/mm^3^, *n* = 12 sections in 3 mice; *p* = 0.696, Mann–Whitney *U* test; Fig. 1*C1*,*C2*). The detected PV^+^ cell densities were also unaltered in the analyzed hippocampal subregions (Fig. 1*C2*). When we quantified percentages of the ErbB4- and PV-coexpressing neurons in the two genotypes, we found that in both genotypes most hippocampal PV^+^ cells coexpressed the ErbB4 receptor (Fig. 1*D1*). Comparing the coexpression results in the entire hippocampus did not show a difference between the genotypes (Fig. 1*D2*). In WT mice, the coexpression covered 77.66% (median, interquartile range 75.85%–86.85%, *n* = 9 slices from 3 mice) of the PV^+^ neurons; in the NRG1^tg-type-I^ mice, it comprised 75.64% (median, interquartile range 72.41%–80.01%, *n* = 12 slices from 3 mice) of the PV^+^ cells (*p* = 0.166, Mann–Whitney *U* test), in agreement with previous studies (Yau et al., 2003; Fazzari et al., 2010; Bean et al., 2014; see also Neddens and Buonanno, 2010). However, when comparing the expression in the hippocampal subareas (Fig. 1*D2*), a significant but small decrease was observed in the coexpression level, specifically in the CA1–2 area (including *alveus*, *stratum oriens*, and *stratum pyramidale*) in the NRG1^tg-type-I^ mice (*p* = 0.043, Mann–Whitney *U* test; Fig. 1*D2*).

These results show that NRG1 type I overexpression does not produce significant changes in the coexpression of ErbB4 and PV in most hippocampal areas or in the spatial distribution of PV^+^ neurons in the hippocampus. Yet, these data suggest that NRG1 overexpression leads to altered ErbB4^+^ cell soma count of interneurons other than those expressing PV. This could either emerge from changes in the migration, survival, and proliferation of these cells during neurodevelopment (Flames et al., 2004; Li et al., 2012a) or be caused by alterations in the expression and trafficking of the receptor (Longart et al., 2007; see Discussion).

We found no significant difference in ErbB4 protein levels between the two genotypes using Western blot analysis of whole-hippocampus extracts (*n* = 6 including 3 males and 3 females in both genotypes, *p* = 0.310, Mann–Whitney *U* test; Fig. 1*E1–E3*). This discrepancy may be attributed partly to the fact that the cell density analysis focused on cells in specific hippocampal subregions, whereas the lysates in the immunoblots comprised the entire hippocampus, possibly masking subregion-specific differences (see Discussion).

In conclusion, the above results suggest that expression level or pattern of the ErbB4 in some hippocampal CA1 and CA3 cells is altered in response to NRG1 type I genomic overexpression (see Discussion). In addition, the analyses confirm earlier findings that ErbB4 is present in the hippocampal interneurons expressing PV or CCK (Vullhorst et al., 2009), and that both PV^+^ and the PV^–^ interneuron subpopulations expressing the receptor ErbB4 are present in the NRG1^tg-type-I^ mouse hippocampus (see Fig. 1*B2*).

### Hippocampal interneurons expressing PV or CCK have reduced synaptic NMDAR-mediated currents in the mice overexpressing NRG1 type I

Next, we studied synaptic AMPAR- and NMDAR-mediated glutamatergic EPSCs in three neuron subpopulations in the CA3 area of acute hippocampal slices; PV^+^ interneurons (Fig. 2*A1*), CCK^+^ interneurons (Fig. 2*A2*), which both commonly express the ErbB4 (see Fig. 1), and pyramidal cells in which the receptor is absent (Vullhorst et al., 2009). All cells were studied in the whole-cell voltage clamp mode in hippocampal slices from mice expressing fluorescent marker (tdTomato) in PV-interneurons (see Materials and methods). The CCK^+^ GABAergic interneurons were identified *post hoc* by positive immunoreaction for cytoplasmic pro-CCK (tested when cell soma was recovered, *n* = 3 in WT control and *n* = 4 in NRG1^tg-type-I^) or axonal CB1R (tested when only interneuron axon was recovered, *n* = 7 and *n* = 7 respectively; Fig. 2*A2*; Katona et al., 1999). We applied electrical microelectrode stimulation in the CA3 *stratum oriens* aiming to activate predominantly associative-commissural fibers. Blockers for GABA_A_ and GABA_B_ receptors (picrotoxin, 100 µm, and CGP55845, 1 µm) were present in all experiments. We found that the NMDAR-mediated EPSCs in PV^+^ interneurons of the NRG1^tg-type-I^ mice were smaller, in comparison to the AMPAR EPSCs, than in their WT littermate controls (measuring a ratio of the NMDAR-EPSC and the AMPAR-EPSC amplitude, N/A ratio; Fig. 2*B1*). The evoked average glutamatergic EPSCs in the NRG1^tg-type-I^ mice were (median, interquartile range): NMDA EPSC, 19.8 pA, 10.4–45.5 pA; AMPAR EPSC, 110.7 pA, 79.1–136.0 pA. Correspondingly, the N/A ratio in the NRG1^tg-type-I^ mice was 0.18, 0.08–0.29 (*n* = 29). In the WT control mice, the NMDA EPSC amplitude was 47.6 pA (median, interquartile range 29.1–60.8 pA), and the AMPAR EPSC amplitude 127.8 pA, 79.6–214.7 pA. Hence the N/A ratio in WT was 0.28, 0.19–0.42 (*n* = 38). The N/A ratios in PV^+^ cells of the two genotypes were different (*p* = 0.010, Mann–Whitney *U* test). Fig. 2*B2* shows cumulative histograms of the N/A ratios measured in the PV^+^ interneurons of the two genotypes.

Likewise, we found that the CCK^+^ CA3 area interneurons in the NRG1^tg-type-I^ mice showed smaller N/A amplitude ratio (median 0.57, interquartile range 0.46–0.98, *n* = 12) than their littermate controls (median 1.12, interquartile range 0.82–1.25, *n* = 10; *p* = 0.019, Mann–Whitney *U* test; Fig. 2*C1*). The EPSC amplitudes in the CCK^+^ interneurons in the NRG1^tg-type-I^ mice were (median, interquartile range): NMDAR EPSC, 31.8 pA, 26.4–43.8 pA; AMPAR EPSC, 54.9 pA, 30.8–65.9 pA (*n* = 12). In the WT CCK^+^ cells, the NMDAR EPSC was 52.6 pA (median, interquartile range 37.1–73.6 pA) and the AMPAR EPSC was 42.5 pA (median, interquartile range 33.2–58.4 pA; *n* = 10). Sample EPSCs in the CCK^+^ interneurons are illustrated in Fig. 2*C1* , and the cumulative histograms of the N/A ratios are shown in Fig. 2*C2*. In line with previous observations, CCK^+^ interneurons had larger NMDAR-mediated synaptic currents (compared as the N/A ratio in the WT mice) than PV^+^ cells (*p* = 0.001, Mann–Whitney *U* test; Maccaferri and Dingledine, 2002; Matta et al., 2013).

In contrast to the interneurons, there was no difference in the N/A ratio across pyramidal cells (PCs) between genotypes (Fig. 2*D1*,*D2*; *p* = 0.761, Mann–Whitney *U* test). EPSCs in the NRG1^tg-type-I^ mice PCs were (median, interquartile range): NMDAR EPSC, 47.5 pA, 34.0–76.2 pA, and AMPAR EPSC, 63.8 pA, 41.9–105.1 pA. Consequently, the N/A ratio in the NRG1^tg-type-I^ mice was 0.80, 0.49–1.11 (*n* = 21). Correspondingly, in the WT mice, the NMDA EPSC amplitude was 73.3 pA (median, interquartile range 45.0–96.5 pA), and AMPAR EPSC amplitude, 100.1 pA (median, interquartile range 49.1–120.2 pA). The N/A ratio in the WT PCs was 0.79, 0.63–0.98 (*n* = 22).

Because both interneuron populations comprise various specialized cell types (Klausberger and Somogyi, 2008; Pelkey et al., 2017), and glutamatergic synapse features may vary between individual interneuron types (Papp et al., 2013), we visualized and anatomically examined the recorded interneurons (filled with neurobiotin) to identify basket cells (PVBCs; Fig. 3*A*) in the PV^+^ subpopulation (see Fig. 2*B*). We confirmed 22 PVBCs (12 in the WT mice and 10 in the NRG1^tg-type-I^ mice). Interestingly, the PVBC group in both genotypes showed parametric distribution of the N/A values (in the NRG1^tg-type-I^ mice *W* = 0.91, *p* = 0.270; in the WT mice, *W* = 0.96, *p* = 0.780; Shapiro–Wilk test) showing that the N/A values have less variation in an identified PV^+^ cell type subpopulation than in the entire PV^+^ cell population in general. The PVBC data showed smaller N/A EPSC ratio in the NRG1^tg-type-I^ mice (0.14 ± 0.04, *n* = 10) than in the WT control mice (0.31 ± 0.04, *n* = 12; *p* = 0.006, mean ± SEM, *t* test; Fig. 3*B1*,*B2*). In addition to the basket cells, we identified two axo-axonic cells (Nissen et al., 2010) in the NRG1^tg-type-I^ mice (their average N/A ratios 0.09 and 0.21) and one in the WT control littermates (N/A ratio = 0.18). Because of their low number, these cells were not separately compared between the genotypes (but the cells were included in the PV^+^ cell pool in Fig. 2).

### Quantal current analysis in parvalbumin basket cells shows unaltered AMPAR-mediated transmission in NRG1^tg-type-I^ Mice

Because the decreased N/A ratio alone is unable to distinguish between suppressed NMDAR currents and increased AMPAR EPSCs, and because altered NRG1 levels can affect AMPAR-mediated transmission (Abe et al., 2011), we next studied glutamatergic miniature currents (mEPSCs) in a new set of identified PVBCs recorded in the CA3 area (Fig. 4*A1*). The cells were voltage clamped at –65 mV for the AMPAR EPSCs and 40 mV for the NMDAR EPSCs in presence of tetrodotoxin (TTX, 1 µm) and the GABA receptor blockers (picrotoxin, 100 µm, and CGP55845, 1 µm). In WT mice PVBCs, the AMPAR-mediated mEPSCs (Fig. 4*A2*) occurred at 3.53 Hz (median, interquartile range 2.32–5.67 Hz, *n* = 7) and had amplitudes of 28.9 pA (median, interquartile range 25.3–32.6 pA, *n* = 7). Correspondingly, in the NRG1^tg-type-I^ mice, the AMPAR mEPSC (Fig. 4*A3*) frequency was 3.28 Hz (median, interquartile range 2.51–3.92 Hz, *n* = 6) and the amplitude 28.3 pA (median, interquartile range 24.8–34.7 pA, *n* = 6). Neither the AMPAR mEPSC frequency (*p* = 0.954) nor the amplitude (*p* = 1.00) differed between genotypes in the PVBCs (Mann–Whitney *U* test).

In addition, we measured the NMDAR-mediated mEPSCs in the same identified PVBCs following wash-in of NBQX (25 μm). One recording was lost before the NBQX application and therefore the *n* number is smaller than above. We found that the frequency of detected NMDAR mEPSCs was not different between WT PVBCs (Fig. 4*A4*; 4.27 Hz, 1.48–6.60 Hz, *n* = 6) and the NRG1^tg-type-I^ PVBCs (Fig. 4*A5*; 2.90 Hz, 2.34–4.33 Hz, *n* = 6; median and interquartile range, *p* = 0.589, Mann–Whitney *U* test; Fig. 4*A6*). Yet, the amplitude of the NMDAR mEPSCs in NRG1^tg-type-I^ mice (19.0 pA, 17.6–20.1 pA; median, interquartile range) was moderately but significantly lower than in WT littermates (21.8 pA, 20.8–30.0 pA; *p* = 0.026, Mann–Whitney *U* test). These results are summarized in Fig. 4*A6*,*A7*.

We also recorded mEPSCs in the CA3 area PCs and found that neither AMPAR- nor NMDAR-mediated mEPSCs differed between the genotypes. In the WT PCs, the AMPAR mEPSC (Fig. 4*B1*) values were 1.15 Hz (median, interquartile range 1.10–1.70 Hz, *n* = 7) and 20.8 pA (median, interquartile range 18.8–21.9 pA, *n* = 7). Correspondingly, in the NRG1^tg-type-I^ mice (Fig. 4*B2*), the values were 0.79 Hz (median, interquartile range 0.54–1.25 Hz, *n* = 10, *p* = 0.223 vs. the WT PCs) and 21.6 pA (median, interquartile range 19.0–25.0 pA, *n* = 10, *p* = 0.354 vs. the WT PCs; Mann–Whitney *U* test). Respectively, the NMDAR mEPSCs in the WT PCs (Fig. 4*B3*) occurred at 2.54 Hz (median, interquartile range 1.60–3.71 Hz, *n* = 5) with amplitude of 18.5 pA (median, interquartile range 17.8–24.1 pA, *n* = 5). The NMDAR mEPSCs in the NRG1^tg-type-I^ PCs (Fig. 4*B4*) occurred at 1.94 Hz (median, interquartile range 1.71–2.87 Hz, *n* = 9, *p* = 0.689 vs. the WT PCs) showing an amplitude of 19.3 pA (median, interquartile range 18.5–20.6 pA, *n* = 9, *p* = 0.790 vs. the WT PCs). The results are summarized in Fig. 4*B5*,*B6*.

The findings of unchanged AMPAR mEPSCs in the PVBCs and PCs (and the moderate reduction of the NMDAR mEPSC amplitude specifically in the PVBCs in the NRG1^tg-type-I^ mice) indicate that the altered N/A ratio observed (Fig. 3) was caused by reduced postsynaptic NMDAR currents in the NRG1^tg-type-I^ mice PVBCs.

### GABAergic inhibitory currents from parvalbumin- or cholecystokinin-expressing CA3 interneurons are not altered in NRG1^tg-type-I^ mice

Given that alterations in NRG1 levels can acutely change inhibitory synapses and modify them long term (Okada and Corfas, 2004; Woo et al., 2007; Chen et al., 2010; Yin et al., 2013; Agarwal et al., 2014), we studied whether GABAergic synaptic output from interneurons expressing either PV or CCK is also altered in the NRG1^tg-type-I^ mice. To selectively stimulate axons from these interneurons, we prepared slices from NRG1^tg-type-I^ and WT mice expressing Cre-protein either in PV^+^ cells or CCK^+^ interneurons and transduced with a Cre-dependent adeno-associated virus (AAV)-channelrhodopsin-2 (ChR2)-eYFP construct (see Materials and methods). Expression of the construct in the two types of GABAergic fibers is illustrated in Fig. 5*A1*,*A2*. GABAergic IPSCs were elicited in the CA3 area pyramidal cells stimulating the interneuron axons locally with brief laser light pulses (3 ms, 473 nm) focused in *stratum pyramidale*. Stimulation intensity was set to use minimal laser power required for stable IPSCs (Fig. 5*B*). In all experiments, the postsynaptic pyramidal cells (voltage clamped at 0 to 10 mV) were recorded in the presence of glutamate receptor blockers NBQX (25 µm) and DL-AP5 (100 µm). The optically evoked IPSCs were blocked with picrotoxin (100 µm) in all experiments tested (*n* = 8 of 8 in IPSCs from PV^+^ fibers, and *n* = 3 of 3 from CCK^+^ fibers).

We found that the IPSCs did not differ significantly between the genotypes for either PV^+^ or CCK^+^ GABAergic synapses (Mann–Whitney *U* test). For PV^+^ fibers, the evoked IPSC amplitudes (Fig. 5*C1*) were 43.6 pA, 34.3–82.0 pA (median, interquartile range; *n* = 18 cells) in WT mice, and 44.8 pA, 36.2–82.7 pA in NRG1^tg-type-I^ mice (*n* = 16 cell, *p* = 0.74 vs. WT; Fig. 5*C2*). The IPSC rise time (from 20% to 80% of the peak) values in the WT were 0.88 ms, 0.76–1.20 ms (*n* = 18 cells), and in the NRG1^tg-type-I^ mice 0.97 ms, 0.87–1.49 ms (*n* = 16, *p* = 0.208 vs. WT; Fig. 5*C3*). The IPSCs from the WT mice PV^+^ fibers showed a decay half-time of 9.4 ms, 8.6–12.4 ms (*n* = 18), and the decay half times in the NRG1^tg-type-I^ PV^+^ cells were 10.1 ms, 9.0–12.8 ms (*n* = 16, *p* = 0.32 vs. WT; Fig. 5*C4*). The paired-pulse ratio (PPR, 50-ms interval, 2nd IPSC/1st IPSC amplitude) in the WT mice was 0.69, 0.60–0.79 (*n* = 18), and in the NRG1^tg-type-I^ 0.58, 0.80–0.76 (*n* = 16, *p* = 0.173 vs. WT; Fig. 5*C5*).

The IPSC amplitudes evoked from the CCK^+^ fibers (Fig. 5*D1*) were 38.0 pA, 23.9–45.4 pA in WT mice (*n* = 5), and 23.4 pA, 18.0–44.7 pA in NRG1^tg-type-I^ mice (*n* = 6, *p* = 0.662 vs. WT; Fig. 5*D2*). The IPSC rise time (from 20% to 80% of the peak) in the WT mice was 2.40 ms, 1.40–3.60 ms (*n* = 5 cells), and 3.30 ms, 1.29–4.30 ms in the NRG1^tg-type-I^ mice (*n* = 5, *p* = 0.94 vs. WT; Fig. 5*D3*). The decay half time in the WT was 7.7 ms, 6.3–12.2 ms (*n* = 5), and in the NRG1^tg-type-I^ mice it was 7.4 ms, 3.4–12.3 ms (*n* = 6, *p* = 0.79 vs. WT; Fig. 5*D4*). The IPSCs evoked from the CCK^+^ fibers showed PPR of 0.61, 0.32–0.66 in WT (*n* = 5), and 0.75, 0.54–1.22 in the NRG1^tg-type-I^ mice (*n* = 6, *p* = 0.33 vs. WT; Fig. 5*D5*).

### Reduced NMDAR-driven recurrent inhibition in the hippocampus in NRG1^tg-type-I^ mice

Finally, we investigated whether the reduced synaptic NMDAR-mediated transmission in these two common recurrent inhibition interneuron subpopulations had consequences for the GABAergic inhibition evoked by repetitive firing of the hippocampal glutamatergic neurons. To study this, we optogenetically stimulated glutamatergic fibers, focusing the laser light pulses in *stratum pyramidale* and *stratum oriens*, aiming to activate the recurrent disynaptic GABAergic pathway. We did the experiments in the CA1 area to avoid polysynaptic glutamatergic discharge generated in the CA3 recurrent glutamatergic circuits (Maccaferri and McBain, 1995). We used hippocampal slices of the NRG1^tg-type-I +/−^ mice and their littermate WT controls both crossed with CaMKII-Cre± and transduced with the AAV-ChR2-eYFP construct in the hippocampus (Fig. 6*A*). We made a translaminar surgical cut in the slices from *alveus* to *stratum lacunosum-moleculare* in the CA1-CA2 area border to exclude the CA3 area recurrent excitatory loop and polysynaptic discharges (Maccaferri and McBain, 1995).

We applied bursts of five pulses of stimuli at 20 Hz every 60 s to generate disynaptic IPSCs in the CA1 area pyramidal cells. The IPSCs were recorded at a reversal potential of the EPSCs (11.1 ± 0.7 mV, mean ± SEM) elicited in the same cells (*n* = 13 comprising 7 cells in the NRG1^tg-type-I^ mice and 6 cells in the WT controls; Fig. 6*B1*,*B2*). Long-term plasticity blockers KN-62 (3 µm) and MCPG (200 µm) were present in all experiments for long-term stability of the disynaptic IPSCs (Perez et al., 2001; Kullmann and Lamsa, 2011; Campanac et al., 2013). After a stable baseline (at least 5 min), NMDAR blocker DL-AP5 (100 µm) was washed in (Fig. 6*C1*,*C2*). This suppressed the evoked recurrent GABAergic IPSC in the WT mice to 0.66 of baseline (charge median, interquartile range 0.61–0.71, *p* = 0.031 vs. baseline, *n* = 6 cells), and in the NRG1^tg-type-I^ mice to 0.74 (charge median, interquartile range 0.66–0.83, *p* = 0.026 vs. baseline, *n* = 7 cells) compared to the baseline (Mann–Whitney rank sum test). The IPSC charge was compared in each experiment between the last 3 min in baseline, and in an equal time window in the presence of DL-AP5 (at 5–8 min after DL-AP5 application). The suppression of the disynaptic IPSCs by the NMDAR blocker was larger in the WT than in the NRG1 mice (*p* = 0.014, Mann–Whitney *U* test). The IPSCs were fully blocked at the end by NBQX (25 µm) in all experiments tested to verify their disynaptic origin (*n* = 4 in the WT controls, and *n* = 4 in the NRG1^tg-type-I^ mice).

The results, summarized in Fig. 6*D*, indicate smaller NMDAR-mediated excitatory drive of hippocampal GABAergic interneurons in the NRG1^tg-type-I^ mice compared to their WT littermates.

## Discussion

Our results show that transgenic overexpression of NRG1 type I, an isoform of NRG1 that has elevated levels in some patients with SZ (Hashimoto et al., 2004; Law et al., 2006; Chong et al., 2008; Weickert et al., 2012; see also Boer et al., 2009; Parlapani et al., 2010; Hahn, 2011), is associated with a hypofunction of NMDAR-mediated synaptic signaling in two major GABAergic interneuron populations in mouse hippocampus.

The reduced ratio of NMDAR- to AMPAR-mediated synaptic currents was observed in the hippocampal GABAergic interneuron populations expressing either PV or CCK, but not in PCs. This finding on cell type specificity is in line with the cortical ErbB4 expression pattern: various studies have demonstrated that ErbB4 expression is predominant in GABAergic interneurons, whereas it is absent in PCs (Vullhorst et al., 2009; Fazzari et al., 2010; Neddens and Buonanno, 2010; Abe et al., 2011; Pitcher et al., 2011; Del Pino et al., 2017). As illustrated in Fig. 1, we confirmed here the ErbB4 expression in both PV^+^ and CCK^+^ interneurons, as has been previously reported (Vullhorst et al., 2009). It should be noted that because of contrast adjustment, Fig. 1*A2* shows low CCK-Cre–dependent fluorophore intensity in the CA3 pyramidal cells compared to interneurons, although CCK is expressed in both cell populations (Burgunder and Young, 1990; Geibel et al., 2014; Rombo et al., 2015).

ErbB4^+^ interneurons expressing either PV or CCK were found in the NRG1^tg-type-I^ mice, but the cell soma counting analysis indicated that the density of ErbB4^+^ neurons not coexpressing PV is reduced in the hippocampus of NRG1 type I–overexpressing mice. This suggests that in some interneurons, either the ErbB4 receptor abundance has changed or detectable ErbB4 immunoreactivity has decreased (e.g. due to an altered subcellular localization or a change in epitope accessibility). Surprisingly, Western blot did not detect the reduction of ErbB4 expression, although the detectable ErbB4^+^ neuron soma number was reduced. We offer two possible explanations for this. The counting of immunohistochemically revealed ErbB4^+^ cell somata is a nonquantitative method (giving cells clearly ErbB4^+^ or cells not confirmed positive). If the antibody-labeled fluorescence signal in the soma is low, it becomes increasingly challenging to confirm it as immunopositive compared to background. This could happen in the NRG1 type I–overexpressing mice without a significant change in total hippocampal ErbB4 protein level, if subcellular location of the ErbB4 changed (decreased in soma) or the ErbB4 protein is internalized in some interneurons (Liu et al., 2007, Longart et al., 2007), making its detection by the antibody less evident. The discrepancy may also be attributed to the fact that the cell density analysis focused on cells in specific hippocampal subregions, whereas the lysates in the immunoblots comprised the entire hippocampus, possibly masking subregion-specific differences.

We found that synaptic NMDAR currents were reduced in interneurons expressing PV or CCK, but not in pyramidal cells in the NRG1^tg-type-I^ mice. Furthermore, we show that not only is the NMDAR-mediated synaptic component reduced in comparison to the AMPAR currents in the CCK^+^ cells or PV^+^ cells, but a similar significant change is also seen in anatomically identified PV^+^ basket cells. The analyses of the quantal miniature currents in identified PV^+^ basket cells indicate that the reduced NMDAR- to AMPAR-mediated synaptic responses are due to smaller postsynaptic NMDAR currents, rather than increased AMPAR EPSCs. Finally, we show reduced NMDAR-dependent excitatory drive of recurrent GABAergic inhibition in the hippocampus of the NRG1 type I–overexpressing mice using optogenetically driven selective stimulation of hippocampal pyramidal cells.

Of note, in this transgenic mouse line, the overexpression of NRG1 type I is under the Thy-1.2 promoter, which is not equally expressed in all hippocampal pyramidal cells (Dobbins et al., 2018). This raises a possibility that NRG1 release in the hippocampus is not homogeneous, having variable effects on ErbB4-positive cells. This might at least partially explain the N/A ratio variation in PV^+^ cells of the NRG1-overexpressing mice illustrated in Fig. 2. However, the N/A ratio variation may also emerge from lack of the NRG1 receptor in some PV^+^ cells and CCK^+^ interneurons (Bean et al., 2014).

The results suggest that NMDAR-signaling abnormalities in these two major GABAergic interneuron populations may contribute to the hippocampal pathophysiology thought to occur in SZ (Harrison et al., 2003; Gonzalez-Burgos et al., 2011; Curley and Lewis, 2012). In this respect, our results bring together three theories of SZ pathophysiology; genetic heritability, inhibitory circuit dysfunction, and NMDAR hypofunction affecting GABAergic inhibitory interneurons such as PV^+^ basket cells (Zylberman et al., 1995; Lisman et al., 2008; Belforte et al., 2010; Korotkova et al., 2010; Lewis et al., 2011; Gonzalez-Burgos and Lewis, 2012; Volk and Lewis, 2014; Banerjee et al., 2015; Krystal, 2015). Malfunction of PV^+^ basket cells has been commonly suggested to underlie aberrant coordinated network activities, in particular the gamma frequency oscillations, is associated with cognitive dysfunction in animal models (Cho et al., 2015), and is hypothesized to do so as well in SZ patients (Buonanno, 2010; Uhlhaas and Singer, 2010; Gonzalez-Burgos and Lewis, 2012; Harrison et al., 2012; Marín, 2012; Nakazawa et al., 2012). Interestingly, the specific alterations of gamma oscillation features that were observed in hippocampal slice preparations from the NRG1^tg-type-I^ mice (Deakin et al., 2012) differed from findings of *in vivo* studies in which NMDARs were selectively knocked out in PV-expressing interneurons (Korotkova et al., 2010; Carlén et al., 2012). In fact, it has been proposed that NMDAR hypofunction in PV^+^ cells renders the brain networks more prone to exhibit the schizophrenia-associated behavioral and electrophysiological alterations, and that the actual phenotypes develop when NMDAR hypofunction simultaneously coexists in other neuron types (Bygrave et al., 2016). Importantly, our results here show postsynaptic suppression of the NMDAR signaling in interneurons expressing CCK. In physiologic conditions, these hippocampal interneurons have large synaptic NMDAR-mediated currents (Fricker and Miles, 2000; Maccaferri and Dingledine, 2002; Matta et al., 2013). Thus, it is likely that the alterations observed in the NRG1^tg-type-I^ mouse hippocampal network activity and hippocampus-dependent behavior (Deakin et al., 2009, 2012) emerge at least partially from NMDAR hypofunction in the PV^+^ and CCK^+^ interneuron subpopulations. Although we failed to detect changes in AMPAR-mediated glutamatergic currents or the function of GABAergic synapses, it is possible that these can be subject to changes at a later stage of the phenotype progression also in the NRG1 type I mutant mice (Woo et al., 2007; Fazzari et al., 2010; Wen et al., 2010; Abe et al., 2011; Ting et al., 2011).

In summary, our results indicate that synaptic NMDAR-mediated signaling in hippocampal interneurons is sensitive to chronically elevated NRG1 type I levels. Further studies will be required to determine the mechanism by which NRG1 type I overexpression results in the observed NMDAR hypofunction, and to what extent these alterations are sufficient to explain the previously reported phenotypes in these mice (Michailov et al., 2004; Deakin et al., 2009, 2012). Possible cellular mechanisms underlying the NMDAR hypofunction include altered receptor subunit phosphorylation (Hahn et al., 2006; Bjarnadottir et al., 2007; Pitcher et al., 2011; Banerjee et al., 2015) or modulation of the trafficking and expression of NMDAR subunits (Ozaki et al., 1997; Gu et al., 2005; Abe et al., 2011; Luo et al., 2014). Importantly, it has been shown that neuregulin 2 (NRG2), which also signals via ErbB4, facilitates the physical interaction of ErbB4 with the NMDAR GluN2B subunit, leading to internalization of the subunit and hence NMDAR hypofunction (Vullhorst et al., 2015). Finally, the changes in NMDAR-mediated synaptic transmission observed in transgenic NRG1 type I mice could in part mirror what takes place in SZ, given the elevated NRG1 type I expression seen in the brain in the disease. Further studies are needed to explore this possibility and the potential role of therapeutic interventions targeting the NRG1 signaling pathway.

## Synthesis

Reviewing Editor: Liset Menendez de la Prida, Instituto Cajal CSIC Decisions are customarily a result of the Reviewing Editor and the peer reviewers coming together and discussing their recommendations until a consensus is reached. When revisions are invited, a fact-based synthesis statement explaining their decision and outlining what is needed to prepare a revision will be listed below.

We all agree your ms has merit and provides new data to the debate on how NRG1 expression controls hippocampal circuit function. We have however some concerns regarding specificity of transgenic lines, that may challenge data interpretation, and several methodological issues that may require further clarification. Also, we would also like you discuss more potential conflicting issues with previous reports on NRG1 function in pyramidal-PV/CCK-Bc microcircuits, and whether methodological issues may affect interpretation (see specific comments below).

Here are main issues raised by reviewers and myself.

R1:

i)The authors used BAC-CCK-cre tg mice to identify CCK+ interneurons in the hippocampus. In the original paper by Geibel et al., 2014, in line with all other CCK-cre lines, CCK expression was reported in pyramidal cells, yet the figure 1 shows no expression of the reporter protein in these neurons only in interneurons. What might be the explanation for this discrepancy?

ii) As the authors know not all CCK+ interneuron express CB1, as e.g. some neurogliaform cells contain CCK but not CB1. Please report how many interneurons were tested for CB1 and how many only for CCK.

iii) The authors applied a high flow rate (5 ml/min) during the recordings. Some explanation for the use of such a high rate might be beneficial.

iv) Miniature events have been recorded and analysed. However, the details of the analysis are not described. Ideally, the same number of events (at least 100 event/cell) should be analysed in each interneuron. Then the parameters obtained in different groups might be statistically compared using Kolmogorov-Smirnov non-parametric test. They may choose to pool all data obtained in a group and then perform the comparison between the two groups.

R2:

1, Based on the western blot result (Figure 1E2), the expression of ErbB4 in female mice looks always higher than that in male mice. Is there any sex-difference in ErbB4 expression? This is an interesting phenomenon that need to be clarified. If that is the case, special attention need to be paid for the rest of experiments since sex would have already affected the outcome of the other experiments.

2, It is unclear why Western-blot did not detect the reduction of ErbB4 expression given that ErbB4+ neuron numbers reduced by almost ~ 43%. The explanation the authors provided is not so convincing because the number of ErbB4+ neurons was evenly reduced in the hippocampus as shown in Figure 1 B2. On the other hand, the representative images (Figure 1 B1) did not show obvious reduction in ErbB4+ neuron numbers in NRG1 overexpression mice. Seems the background fluorescence in WT image is higher than that in transgenic mice, which might affect the counting of cell number.

3, For Figure 1A, it is unclear how many ErbB4+ neurons are PV+/CCK+ and vice versa. Based on a previous report (J. C. Bean, Journal of Neuroscience, 2014), 29.8% ErbB4+ neurons are PV+ and 71% PV+ neurons are ErbB4+ in the hippocampus. So not all PV+ neurons are ErbB4+, the same condition should also apply to CCK+ neurons. Thus, data presented in Figure 2 may contain some PV+/CCK+ but ErbB4- neurons.

4, The interneurons in the hippocampus are very heterogeneous. The statement that “... PV and CCK, the mutually exclusive neuronal markers which together label the majority of the hippocampal interneuron populations ...” is not true. Actually, PV and CCK only label peri-somatic interneurons which represent parts of interneurons in the hippocampus.

5, Thy-1.2 is preferentially expressed in projection neurons, but the hypofunction of NMDA receptor was only found in interneurons in the transgenic mice. Thus, the effects of overexpressed NRG1 which is driven by Thy-1.2 promoter should be through non cell-autonomous mechanisms. However, it is still interesting to see if NRG1 overexpression has any cell-autonomous effects on pyramidal neurons. Such as resting membrane potential, input resistance, neuronal excitability etc.

6, Typos:

‘signalling’ in the title and main text should be ‘signaling’.

Page 2 line 16, ‘co-localised’ should be ‘co-localized’.

Page 5 line 73, ‘behavioural’ should be ‘behavioral’.

Page 23 line 534, ‘utilising’ should be ‘utilizing’.

AE comments:

- Using Thy-1.2 as a promoter leads to NRG1 overexpression in a subset of pyramidal cells (presumably at deep CA1 layers). We and others recently found different connectivity between deep and superficial CA1 pyramidal cells and PV+ and CCK+ BCs(See Lee et al Neuron 2014; Valero et al. Nat Neu 2015). Given potential differences with previous results on NRG1-ErbB4 (Fazzari et al; del Pino; Ting et al, etc..) one wonders whether hypofunction of NMDAr may be subcircuit-dependent.

- Authors should discuss more carefully conflicting results in the field.

- Data from CA1 and CA2 were pooled together (see Fig.1). Given the role of CA2 in schizophrenia and specific forms of plasticity as compared with CA1 (see work by Siegelbaum, Chevalyere, Piskorowski) authors should consider to present data separately and/or discuss this point further.

- Please, note that PV and CCK at least in the hippocampus are expressed not only in perisomatic inhibitory cells but also in dendrite targeting interneurons (e.g. PV in bistratified, CCK in PPA cells). Check carefully your statements
